# *Fasciola hepatica* glycoconjugates immuneregulate dendritic cells through the Dendritic Cell-Specific Intercellular adhesion molecule-3-Grabbing Non-integrin inducing T cell anergy

**DOI:** 10.1038/srep46748

**Published:** 2017-04-24

**Authors:** Ernesto Rodríguez, Hakan Kalay, Verónica Noya, Natalie Brossard, Cecilia Giacomini, Yvette van Kooyk, Juan J. García-Vallejo, Teresa Freire

**Affiliations:** 1 Laboratorio de Inmunomodulación y Desarrollo de Vacunas, Departamento de Inmunobiología, Facultad de Medicina, Universidad de La República, Montevideo, Uruguay; 2 Department of Molecular Cell Biology and Immunology, VU University Medical Center, Amsterdam, The Netherlands; 3 Laboratorio de Bioquímica, Departamento de Biociencias, Facultad de Química, UdelaR, Montevideo, Uruguay

## Abstract

Dendritic cell-specific ICAM-3 grabbing non-integrin (DC-SIGN) expressed on a variety of DCs, is a C-type lectin receptor that recognizes glycans on a diverse range of pathogens, including parasites. The interaction of DC-SIGN with pathogens triggers specific signaling events that modulate DC-maturation and activity and regulate T-cell activation by DCs. In this work we evaluate whether *F. hepatica* glycans can immune modulate DCs via DC-SIGN. We demonstrate that DC-SIGN interacts with *F. hepatica* glycoconjugates through mannose and fucose residues. We also show that mannose is present in high-mannose structures, hybrid and trimannosyl N-glycans with terminal GlcNAc. Furthermore, we demonstrate that *F. hepatica* glycans induce DC-SIGN triggering leading to a strong production of TLR-induced IL-10 and IL-27p28. In addition, parasite glycans induced regulatory DCs via DC-SIGN that decrease allogeneic T cell proliferation, via the induction of anergic/regulatory T cells, highlighting the role of DC-SIGN in the regulation of innate and adaptive immune responses by *F. hepatica*. Our data confirm the immunomodulatory properties of DC-SIGN triggered by pathogen-derived glycans and contribute to the identification of immunomodulatory glyans of helminths that might eventually be useful for the design of vaccines against fasciolosis.

C-Type lectin receptors (CLRs) on dendritic cells (DCs) recognize a broad range of pathogens through the interaction of specific carbohydrate signatures that they express/produce^1^. They also regulate signaling or cellular interactions such as DC migration and T cell binding, modulating both innate and adaptive immunity^1^. Different CLRs have been identified as critical players in the carbohydrate-mediated immuneregulation induced by pathogens, such as MR, MGL, Dectin, or DC-SIGN. The latter, also known as ICAM-3 grabbing non-integrin or CD209, is a type II transmembrane protein that binds mannose- or fucose-containing N-glycans^2^. Previous works have demonstrated that DC-SIGN interacts with glycan structures from a variety of pathogens ranging from virus to fungi^3^. In fact DC-SIGN binds glycoconjugates from HIV^4^, coronavirus^5^, herpes simplex virus^6^, *Helicobacter pylori*^4^, *Mycobacterium tuberculosis*^4^,^7^,^8^, *Candida albicans*^9^, as well as cancer-related glycans^10^. More recently, different papers have reported that DC-SIGN also interacts with glycans form parasites, thereby modulating the immune response. Indeed, DC-SIGN recognizes not only protozoan parasites such as *Leishmania* sp.^11^, but also helminth parasites, such as *Schistosoma mansoni*^12^,^13^,^14^ or *Trichuris suis*^15^.

DC-SIGN is abundantly expressed on a variety of DCs, including monocyte- and CD34^+^-derived DC, as well as *in vivo* dermal DC of the skin and immature DC of both peripheral and lymphoid tissues. The interaction of DC-SIGN with pathogens, triggers specific signaling events that modulate DC activity at various levels, influencing phagocytosis^16^, suppressing TLR-induced maturation of DCs^15^, internalizing pathogen-derived molecules^12^, modifying DC-adhesion and migration^17^ and antigen presentation^18^,^19^,^20^, and regulating T-cell activation by DCs^4^,^7^. In addition, DC-SIGN has also been involved in the induction of anti-inflammatory signals that result in the induction of anergic T cells^21^. Interestingly, it has been recently reported that extracts from *S. mansoni* eggs and *F. hepatica* worms trigger a DC-SIGN specific signaling pathway on DCs that directs differentiation of T cells into follicular helper T cells^7^.

To allow long survival in their hosts, helminth parasites evade host immunity by altering DC maturation and function^15^,^22^,^23^,^24^, resulting in Th2 polarization. Fasciolosis, a helminth infection caused by *Fasciola hepatica*, is of paramount importance due to its wide spectrum of definitive hosts^25^ and its worldwide distribution^26^ affecting both cattle and human health. Several studies have independently demonstrated that *F. hepatica*-derived molecules inhibit or decrease DC activation, which results in the induction of a tolerogenic phenotype^27^,^28^,^29^,^30^. In addition, *F. hepatica* tegumental antigen modulates DC activity by suppressing MAPK-signaling and by up-regulating the expression of the suppressor of cytokine signaling 3 (SOCS3)^31^. Furthermore, we have demonstrated that DCs from mice infected with *F. hepatica* have a semi-mature phenotype that is characterized by low MHC II and CD40 expression and high secretion of the immunoregulatory cytokine IL-10^32^. Thus, it has been hypothesized that *F. hepatica* may modulate DC function and fate as a mean to control its pathogenesis and survival in the infected hosts.

Very recent reports^33^,^34^, including our own^32^, have brought insights about the role of *F. hepatica* glycans in mediating the regulation of DC-maturation through CLR recognition. Our group has recently described that glycan structures produced by *F. hepatica* participate in the modulation of bone marrow-derived DCs (BMDCs) and induce/mediate the production of IL-10 and IL-4 during infection^32^. Moreover, mannose inhibition indicated that a mannose-specific receptor mediates the recognition of *F. hepatica* glycans by DCs^32^. More recently, the mannose receptor was found to mediate parasite tegumental glycan recognition by BMDCs^33^,^34^, although further experiments suggested that also other mannose-specific CLRs are participating in *F. hepatica* modulation of DCs^34^. Last, a MR-dependent mechanism of inducing T cell anergy by DCs loaded with parasite tegumental molecules was reported^33^. Yet, molecular mechanisms associated to the modulation of human DC function by *F. hepatica* are scarce.” Thus, we sought to evaluate whether *F. hepatica* glycoconjugates interact with DC-SIGN and whether this interaction regulates the stimulatory function of DCs by evaluating their capacity to induce regulatory or anergic T cells.

In this work we show that *F. hepatica* glycans on human DCs induce a strong production of TLR-induced IL-10 and IL-27p28 in a process that requires interaction with DC-SIGN. Since DC-SIGN has been shown to bind Man and Fuc, and these glycans are recognized by DC-SIGN on FhTE, it is highly suggestive that they mediate DC-SIGN effect. In addition, these glycans induce regulatory monocyte-derived DCs (mo-DCs) via DC-SIGN that decrease allogeneic T cell proliferation, highlighting the role of DC-SIGN in the regulation of innate and adaptive immune responses by *F. hepatica*. Our data confirm the immunomodulatory properties of DC-SIGN triggered by *F. hepatica*-derived glycoconjugates and contribute to the deep understanding of the immunoregulation strategies by this helminth.

## Results

### DC-SIGN uptakes *F. hepatica* glycoconjugates and mediates the enhanced production of TLR-induced IL-10 and IL-27p28 by mo-DCs

**Figure 1:**
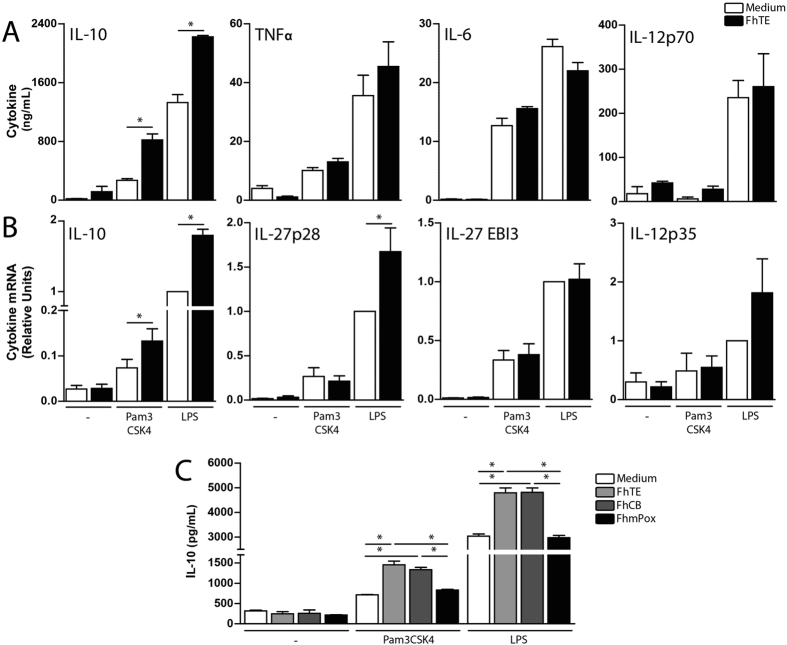
*F. hepatica* glycoconjugates favor the production of IL-10 by TLR-triggered mo-DCs. (**A**) IL-6, IL-10, TNFα and IL-12p70 levels determined by ELISA on supernatants from Pam3CSK4- and LPS-stimulated mo-DC cultures incubated with and without FhTE. (**B**) IL-10, IL-27p28, IL-27 EBI3 and IL-12p35 levels determined by qRT-PCR of purified RNA of Pam3CSK4- and LPS-sitmulated mo-DC cultures incubated with and without FhTE. (**C**) IL-10 levels determined by ELISA on supernatants of TLR-triggered mo-DCs incubated in the presence of FhTE, FhCB (oxidation negative control) or FhmPox (oxidized FhTE). A representative Figure of four independent experiments is shown (±SD, indicated by error bars). Asterisks indicate statistically significant differences (*p* < 0.05).

To determine whether *F. hepatica* glycans modulate DC-mediated immune responses, we first evaluated whether a total preparation of parasite components (FhTE) can modulate the production of cytokines induced after a maturation stimulus by DCs. To this end, immature mo-DCs were cultured in the presence or absence of Pam3CSK4 or LPS and FhTE, and the production of different cytokines were evaluated in the culture medium. Although FhTE alone did not induce the expression of cytokines by mo-DCs, when cultured together with a maturation stimulus, it enhanced the production of IL-10 and IL-27p28 by mo-DCs (Fig. 1A and B). Interestingly, this enhanced production of IL-10 was abrogated when FhTE glycans were oxidized with meta-periodate (FhmPox), a common method used to evaluate the biological activity of glycans (Fig. 1C)^32^. These results indicate that *F. hepatica* glycoconjugates mediate the enhanced production of TLR-induced IL-10 and IL-27p28 by mo-DCs.

**Figure 2:**
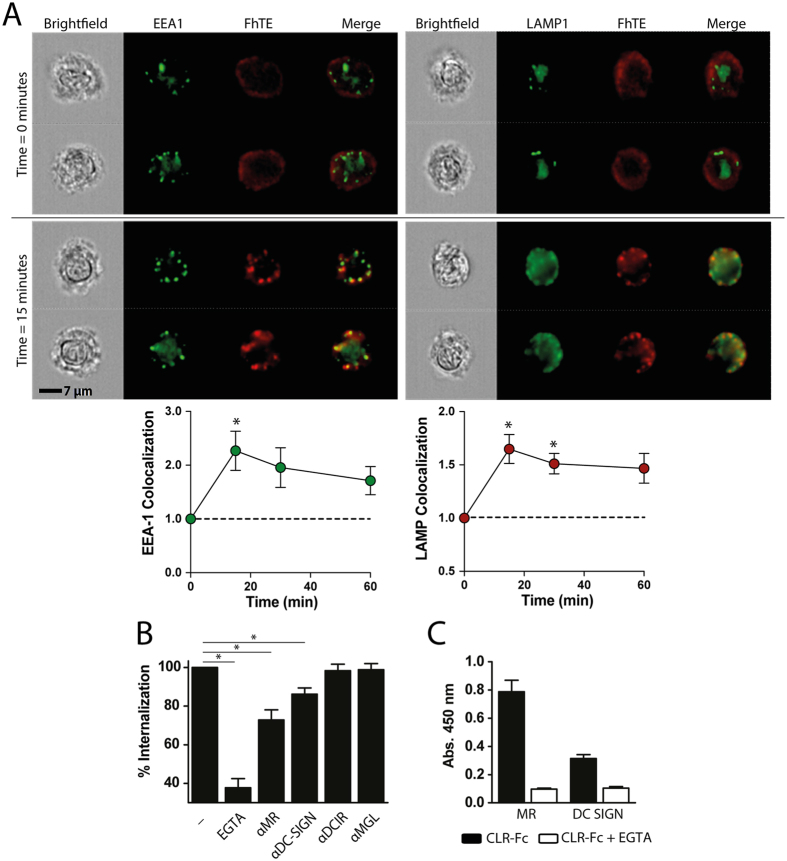
*F. hepatica* glycoconjugates are uptaken by MR and DC-SIGN. To evaluate FhTE-uptake by mo-DCs, cells were incubated with Alexa 647-labeled FhTE at 37 °C. **(A**) Internalization of FhTE was followed in time and co-localization scores with the routing markers EEA1 and LAMP1 were calculated using imaging flow cytometry. Plotted values were normalized to the condition obtained at 0 min. (**B**) To identify the receptors that mediate the internalization, moDCs were incubated with Alexa 647-labeled FhTE for 1 h at 37 °C or 4 °C as a control in presence of EGTA, MR-, DC-SIGN, DCIR- and MGL-specific antibodies, and analyzed by FACS. Internalization was calculated as the difference between the MFI at 37 °C and MFI at 4 °C. (**C**) MR and DC-SIGN bindings were evaluated on FhTE-coated plates with MR-Fc and DC-SIGN-CLR in presence or absence of EGTA. A representative Figure of four independent experiments is shown (±SD, indicated by error bars). Asterisks indicate statistically significant differences (*p* < 0.05).

Next, we investigated whether parasite components are taken up by mo-DCs. To this end we studied the intracellular routing of FhTE components by imaging flow cytometry. As shown in Fig. 2A, the localization of labeled-FhTE molecules was intracellular and they co-localized at 15 minute-incubation period with early endosomal antigen (EEA-1) and the lysosomal marker LAMP-1. Uptake of labeled-FhTE to moDCs was also confirmed by flow cytometry and inhibited upon incubation with EGTA (Fig. 2B), suggesting the participation of a CLR in this process. Furthermore, FhTE-uptake to mo-DCs was inhibited with MR- and DC-SIGN-specific antibodies, while it was not modified when using anti-DCIR and anti-MGL antibodies (Fig. 2B). Finally, we evaluated the capacity of MR and DC-SIGN to recognize coated-FhTE using MR- and DC-SIGN-Fc constructions, confirming their participation in the recognition of glycans present in FhTE, which was inhibited with the chelating agent EGTA (Fig. 2C).

**Figure 3:**
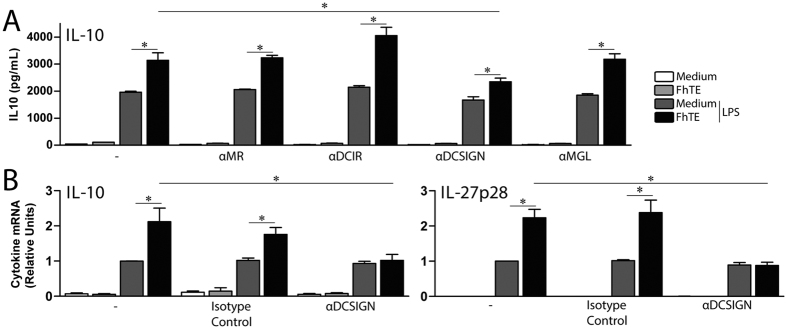
DC-SIGN favors the production of IL-10 and IL-27p28 by LPS-stimulated mo-DCs. (**A**) IL-10 production levels on supernatants from LPS-stimulated mo-DC overnight cultures incubated with and without FhTE in presence of MR-, DC-SIGN, DCIR- and MGL-specific antibodies. (**B**) IL-10 and IL-27p28 production on LPS-stimulated mo-DC cultures incubated with and without FhTE together with anti-DC-SIGN antibodies or isotype control. A representative Figure of four independent experiments is shown (±SD, indicated by error bars). Asterisks indicate statistically significant differences (*p* < 0.05).

Given the capacity of MR and DC-SIGN to recognize and internalize FhTE we investigated whether these receptors mediated the enhanced production of TLR-induced IL-10 and IL27-p28 by mo-DCs stimulated with FhTE. Mo-DCs were incubated with FhTE and LPS in presence of MR-, DC-SIGN-, DCIR- and MGL-specific blocking antibodies. As shown in Fig. 3, only the anti-DC-SIGN antibody was capable of restoring IL-10 levels produced by LPS-stimulated mo-DCs. In addition, the DC-SIGN blocking antibody also restored the levels of IL-27p28 (Fig. 3B) suggesting that DC-SIGN and not MR mediates the modulation triggered by FhTE together with a TLR ligand.

### DC-SIGN interacts with *F. hepatica* Man and Fuc residues present on FhTE

**Figure 4:**
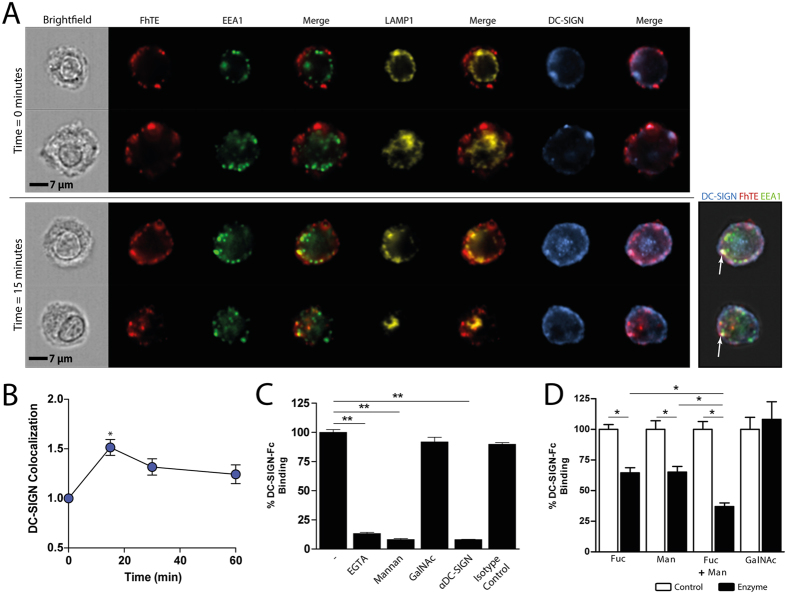
DC-SIGN on mo-DCs interacts with FhTE via Man and Fuc residues. (**A**) Analysis of FhTE internalization using Imaging Flow Cytometry. The co-localization of Alexa 647-labeled FhTE, EEA1, LAMP1 and DC-SIGN was studied. (**B**) Co-localization scores of Alexa 647-labeled FhTE with DC-SIGN were calculated over time using imaging flow cytometry. Plotted values were normalized to the condition obtained at 0 min. (**C**) DC-SIGN binding was evaluated on FhTE-coated plates with DC-SIGN-Fc in presence of EGTA, mannan, GalNAc, anti-DC-SIGN antibody or isotype control. (**D**) DC-SIGN binding was evaluated on Fucosidase (Fuc), mannosidase (Man) or GalNAcase (GalNAc) treatments of FhTE with DC-SIGN-Fc. A representative Figure of four independent experiments is shown (±SD, indicated by error bars). Asterisks indicate statistically significant differences (*p* < 0.05).

Since DC-SIGN showed to participate both in the recognition of *F. hepatica* glycans and in the regulation of TLR-induced IL-10 and IL-27p28 production by DCs, we analyzed in detail the interaction between DC-SIGN and FhTE by imaging flow cytometry. Results on Fig. 4 demonstrate that labeled-FhTE interacted with DC-SIGN on the cell surface (Fig. 4A, time 0) and co-localized with DC-SIGN also at an early time-point (Fig. 4A and B). Interestingly, after 15 minutes of incubation, co-localization of FhTE with both DC-SIGN and EEA1 was observed (shown by white arrows in Fig. 4A), but not with LAMP-1. These data suggest that DC-SIGN-mediated FhTE-uptake by mo-DCs could lead to the delivery of parasite glycoconjugates into early endosomes.

In order to characterize the nature of the carbohydrates recognized by DC-SIGN, we carried out two different approaches: binding inhibition with carbohydrates and treatment with exoglycosidases. The interaction between DC-SIGN-Fc and coated-FhTE was inhibited with incubation with EGTA, mannan and the DC-SIGN-specific blocking antibody, while no inhibition was detected with GalNAc or the isotype antibody control (Fig. 4D). Considering that DC-SIGN can recognize both mannosylated and fucosylated glycans, we further evaluated its binding to FhTE when treated with mannosidase, fucosidase, or both enzymes. Treatment of FhTE with any of these glycosidases inhibited DC-SIGN binding (40% inhibition), while treatment of FhTE with both fucosidase and mannosidase resulted in a 70% inhibition rate (Fig. 4D), indicating that DC-SIGN interacts with both fucosylated and mannosylated parasite glycans. GalNAc-ase treatment of FhTE was used as control, revealing no changes in DC-SIGN binding to FhTE as expected due to the lack of MGL binding observed in Fig. 2D.

Characterization of the N-glycan binding specificity of DC-SIGN has revealed that DC-SIGN may recognize complex-type N-linked glycans expressing Fucα1-3GlcNAc moieties and in lesser extent oligomannose-type *N*-glycans^35^. To determine the mannosylated and fucosylated N-glycan moietes present in FhTE that may act as DC-SIGN ligands, we characterized and quantified the parasite N-glycans by glycan nanoprofiling^36^,^37^. Our analyses showed that the N-glycans present in FhTE contained mainly high mannose structures, with only few hybrid and complex glycans with terminal GlcNAc (Supplementary Fig. S1). These mannosylated structures constitute potential ligands for DC-SIGN. Alpha-1,6-fucosylated paucimannose glycans were also detected. However, since fucosylation was only identified in the common core structure unit (Supplementary Fig. S1), these structures would not represent potential ligands of DC-SIGN because it recognizes glycans that are terminally fucosylated such as LDNF, Le^x^ and pseudo-Le^y^ 35,38.

### *F. hepatica* glycoconjugates induce regulatory mo-DCs via DC-SIGN that decrease allogeneic T cell proliferation

**Figure 5:**
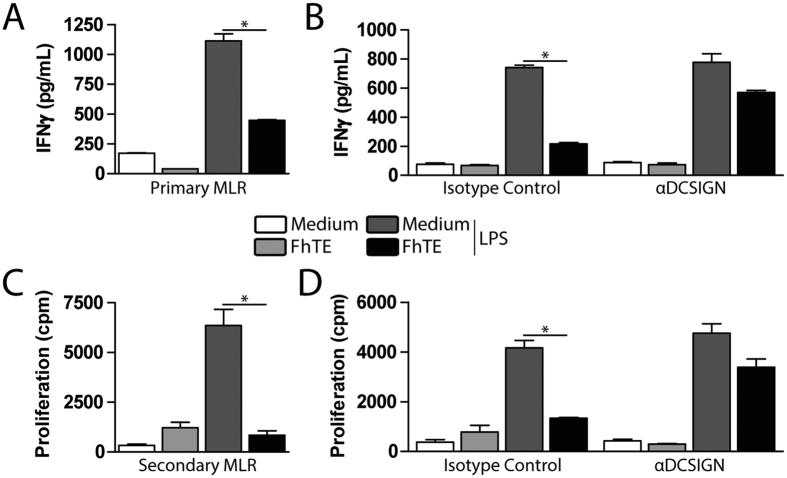
Simultaneous DC-SIGN and TLR4 triggering by FhTE and LPS on mo-DCs decreases allogeneic T cell proliferation. (**A**) Mo-DCs were incubated overnight with FhTE with or without LPS (10 µg/ml) and exposed to allogeneic naïve CD4^+^ T cells. IFN-γ was analyzed by ELISA. (**B**) The same experiment in presence of DC-SIGN specific antibodies and isotype control. (**C**) T cells were allowed to rest, and then restimulated with LPS-matured DCs. T-cell proliferation was measured by ^3^H-Thymidine incorporation in presence or absence of DC-SIGN specific antibodies and isotype control. A representative Figure of four independent experiments is shown (±SD, indicated by error bars). Asterisks indicate statistically significant differences (*p* < 0.05).

To further investigate whether the immune modulation induced by FhTE on LPS-matured mo-DCs affected their T-cell stimulatory function we incubated FhTE-treated mo-DCs in presence or absence of LPS with allogeneic CD4^+^ T cells. Both T cell proliferation and the production of IFNγ were significantly reduced when LPS-stimulated mo-DCs were incubated in presence of FhTE (Fig. 5A). Moreover, the production of IFNγ was partially restored when using the anti-DC-SIGN blocking antibody, suggesting that FhTE regulates the stimulatory capacity of mo-DCs in a process mediated, at least in part, by DC-SIGN (Fig. 5B) and *F. hepatica* mannose-rich glycans.

**Figure 6:**
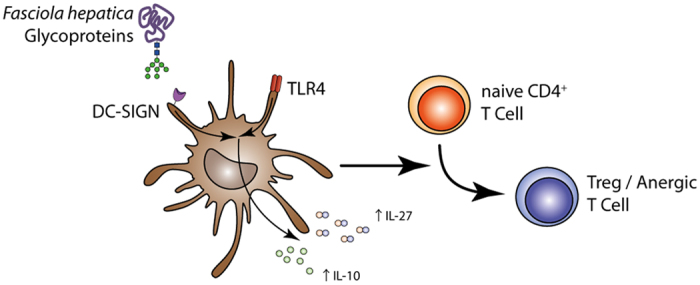
Schematic illustration summarizing the main findings in the present study. Fucosylated and mannosylated glycoconjugates on *F. hepatica* interact with DC-SIGN expressed on the DC surface, triggering a tolerogenic program together with TLR signaling that induces enhanced expression of IL-10 and IL-27 by DCs and differentiates naïve CD4^+^ T cells into anergic/regulatory T cells.

Next, we investigated the effect of FhTE exposure to DCs on T cell proliferation in a secondary MLR. CD4^+^ T cells exposed to DCs pulsed with FhTE showed decreased capacity to proliferate in response to a second stimulation with fully activated DCs (Fig. 6C). This was interpreted as the induction of anergic T cells or T reg cells during the primary MLR. Interestingly, the induction of anergic or regulatory T cells by FhTE-stimulated mo-DCs was mediated by DC-SIGN, since the incubation with the DC-SIGN-specific blocking antibody restored the T cell proliferation (Fig. 6D). These findings illustrate that mannose glycans on FhTE that interact to DC-SIGN on DCs can induce tolerance under inflammatory conditions through the induction of IL-10 and T cells with suppressor capacity.

## Discussion

This study provides evidence that *F. hepatica* glycans modulate human DCs through DC-SIGN, favoring the induction of Tregs or cell anergy. In particular, we show that oligomannose glycans present on FhTE preparation enhance the TLR-induced production of IL-10 and IL-27p28. Although *F. hepatica* glycoconjugates were both recognized and internalized by MR and DC-SIGN on DCs, the observed immunomodulation of TLR-matured DCs was mediated only by DC-SIGN and not by MR, DCIR or MGL, suggesting a main role of this receptor in the DC-immunoregulatory properties induced by this helminth. DC-SIGN interacts with a wide range of pathogens of major impact on public health, including viruses^4^,^5^,^6^, bacteria^8^,^30^ and parasites^11^,^12^,^13^,^14^ via both fucose (for instance in Lewis and sulfo-Lewis structures) and high-mannose glycans in a Ca-dependent manner^39^. However, until now, DC-SIGN interaction with helminths was reported only via fucosylated glycans and not high-mannose structures^14^,^40^. Oligomannose and paucimannose N-glycans present in FhTE could constitute ligands for DC-SIGN through mannose. Our results demonstrate the capacity of DC-SIGN to recognize mannosylated glycoconjugates on *F. hepatica* components, since the binding of DC-SIGN to FhTE was partially abolished after mannosidase treatment. In addition, we also confirm that DCs interact with helminth fucosylated-glycans via DC-SIGN. Considering that we did not identify potential fucosylated N-glycans that could serve as ligands for DC-SIGN, and together with the fact that defucosylation of FhTE reduces DC-SIGN recognition for parasite components, we propose that DC-SIGN fucosylated ligands are present in O-glycans or glycolipids. Of note, since FhTE is a total parasite lysate, it contains both external and internal glycoconjugates. Thus, to establish the role of DC-SIGN during *F. hepatica* infection, *in vivo* studies are necessary.

*F. hepatica* evades host immunity by different mechanisms, including immunomodulation of DC maturation and function^27^,^30^,^32^, resulting in. altered Th2 polarization and inducing tolerogenic/regulatory properties^28^. We have previously reported the expression of IL-10 by semi-matured DCs from *F. hepatica* infected animals^32^, indicating that the parasite is able to immune-regulate DCs during infection. We now demonstrate that *F. hepatica* glycans can immunomodulate TLR-induced maturation of human DCs in a DC-SIGN-dependent process. Thus, DC-SIGN interacts with *F. hepatica* glycans and mediates the increased production of IL-10 and IL-27p28 by TLR-matured DCs. IL-10 is a key immune-regulatory cytokine that can impair the proliferation, cytokine production and migratory capacities of effector T cells^41^ or induce T cell anergy^42^. On the other hand, IL-27 can both exert immuno-stimulatory or suppressive effects. Interestingly, IL-27 can induce IL-10 production and induce the differentiation of the IL-10-producing type 1 regulatory T (Tr1) cells^43^. In agreement with our results, fucose-induced triggering of DC-SIGN resulted in the induction of IL-27p28 expression and secretion by DCs, driving subsequent follicullar T helper polarization^7^. Furthermore, this type of phenotype on DCs is induced by other helminths, such as *S. mansoni*^1^,^4^,^44^, suggesting that fucosylated glycans present on FhTE would trigger DC-SIGN-signaling. In addition, our results indicate that DC-SIGN recognizes next to fucosylated glycans also mannosylated glycans, which can both be present in parasites, that may have a different functional consequence increasing IL-10 and T regs. Additional experiments are needed to determine whether both glycan moieties in *F. hepatica* glycans trigger different DC-SIGN signaling pathways.

CLR-triggering has been correlated with regulatory T cell differentiation in different pathological contexts^44^. In particular, DC-SIGN can favor Th1/Th17, Th2, follicular T helper or regulatory T cell polarization^44^. To further understand whether *F. hepatica*-triggering of DC-SIGN on DCs can alter T cell activation, we evaluated whether TLR-matured DC conditioned with parasite components were able to limit T cell proliferation. Our results indicate that human DC-SIGN promotes a tolerogenic/regulatory state on DCs since *F. hepatica*-conditioned DCs induced regulatory mo-DCs via DC-SIGN that decrease allogeneic T cell proliferation by the induction of induction of T-cell tolerance. On the other hand, the interaction of myelin oligodendrocyte glycprotein (MOG) with DC-SIGN in the context of simultaneous TLR4 activation resulted in enhanced IL-10 secretion and decreased T cell proliferation^21^. Furthermore, *H. pylori* glycans that interact with DC-SIGN induced tolerogenic DCs that failed to induce T cell effector functions but induced regulatory T cells^45^. In the context of *F. hepatica* immunoregulatory properties, other CLRs can also participate in the induction of T cell anergy as has been recently proposed for the MR on bone marrow-derived DCs induced anergic-like T cells via DCs, although only a small reduction of T cell anergy was found when using DCs from knock-out mice, suggesting that other CLRs can participate in this process^33^. Furthermore, this process did not seem to be mediated by recognition of parasite glycoconjugates by the MR on BMDCs but the MR interacting with CD4^+^ T cells^33^. Thus, our work provides an additional mechanism of induction of T cell anergy induced by *F. hepatica*-conditioned DCs.

In conclusion, our work demonstrates that *F. hepatica* fucosylated and mannosylated glycoconjugates are recognized by DC-SIGN, which in turn triggers a tolerogenic program on TLR-matured DCs in presence of parasite glycosylated molecules characterized by the increased production of IL-10 and IL-27 and confer DCs with the capacity to induce regulatory/anergic T cells (Fig. 6). These results highlight the biological role of glycans in the immune evasion mechanisms triggered by helminths.

## Methods

### Ethics statement

Human monocytes were isolated from buffy coats of healthy donors in accordance with guidelines and regulations of the Ethical Committee of the VU University Medical Center (Amsterdam, Netherlands). Informed consent was given by all donors for the use of their blood samples. Adult worms were collected during the routine work of a local abattoir (Frigorífico Carrasco) in Montevideo (Uruguay). Protocols were approved by the Uruguayan Committee on Animal Research (Comisión Honoraria de Experimentación Animal, Uruguay) or by the Ethical Committee of the VU University Medical Center (Amsterdam, Netherlands).

### Preparation of protein lysates from *F. hepatica*

Live adult worms of *F. hepatica* were obtained from the bile ducts of bovine livers, washed in phosphate buffered saline (PBS) pH 7.4, then mechanically disrupted and sonicated. After centrifugation at 40,000 × *g* for 60 min supernatants were collected and dialyzed against PBS. The obtained total lysate (FhTE) was quantified and stored at −80 °C. Carbohydrate glycol groups present in FhTE were oxidized with sodium periodate (10 mM). The oxidation was performed at room temperature for 45 min in the dark, followed by the reduction with sodium borohydride (50 mM) of the reactive aldehyde groups. The resulting oxidized lysate is referred as FhmPox. In order to perform control experiments, the following control extracts were prepared: FhCB, consisted of FhTE subjected to the whole treatment excepting for the incubation with sodium periodate; and CmPox, consisting of PBS subjected to the entire treatment. Lysates were dialyzed against PBS and their protein concentration was measured using the bicinchoninic acid assay (Sigma-Aldrich, St. Louis, MO). The endotoxin levels were determined by using the Limulus Amebocyte Lysate kit Pyrochrome (Associates of Cape Cod). Protein preparations showed very low levels of endotoxins and were not able to induce DC-maturation (as IL-12 read out) on their own. mo-DCs incubated with the control CmPox produced the same levels of cytokines in the culture supernatant as mo-DCs cultured in medium alone.

### Monocyte derived dendritic cells (moDCs)

Monocytes were isolated from peripheral blood mononuclear cells (PBMCs) from buffy coats of healthy donors (Sanquin, The Netherlands) by a lymphoprep gradient (Axis-Shield, San Diego, CA) and subsequent percoll gradient centrifugation (GE Healthcare Life Science, Netherlands). Informed consent was obtained from all blood donors for the use of their blook samples. DCs were generated by culturing purified monocytes in complete medium consisting of RPMI 1640 (Thermo Fisher Scientific, Netherlands) supplemented with 10% fetal bovine serum (BioWhittaker), 1000 U/ml penicillin/streptomycin (Lonza, Netherlands), and 2 mM glutamine (Lonza, Netherlands) in combination with IL-4 (262.5 U/ml; Biosource, Belgium) and GM-CSF (112.5 U/ml; Biosource, Belgium) for 4–7 days.

For DC-maturation assays, moDCs (2 × 10^5^/well) were incubated at 37 °C and 5% CO_2_ in 96-well plates with plate-bound FhTE, FhmPox or FhCB (125 μg/ml) in the presence or absence of Pam2CysK4 (TLR2, 10 μg/ml). When appropriate, DCs were pre-incubated for 2 hrs at 37 °C with the blocking anti-DC-SIGN (AZD-N1), -DCIR (111F8.04,), MR (19.2), -MGL (1G6.6), antibodies. IL-6, IL-10, IL-12p70 and TNF-α levels were determined by specific ELISAs (eBiosciences, CA or BioSource, Belgium) after overnight incubation. Alternatively, IL-10, IL-27p28, IL-29EBI3 and IL-35 were determined by quantitative real time RT-PCR (qRT-PCR) after a stimulation of 6 hrs.

### T regulatory cell/anergy induction assays (*MLRs*)

FhTE/LPS-stimulated mo-DCs were washed and incubated for 6 days with allogeneic naive CD4^+^CD45^+^ T cells that were previously purified by negative selection (Invitrogen). Primed T cells were recovered, washed, and allowed to rest for 3 d in the presence of IL-7 and IL-15 (ImmunoTools). After 9 d of the primary stimulation, primed T cells were washed and restimulated by co-culturing them with LPS-activated moDC from the original donor at different ratios. After 6 d, plates were pulsed with [^3^H]thymidine (1 μCi/well; GE Healthcare) for an additional 18 h and [^3^H]thymidine incorporation was measured on a Betaplate 1205 liquid scintillation counter (Wallac-LKB).

### Real-time PCR

Total RNA was isolated from DC cultures, by use of Tri-reagent (Sigma-Aldrich). Real-time PCR were set up using the SYBR Green method in an ABI 7900HT sequence detection system as previously described^46^. Supplementary Table 1 shows a list with the primers used.

### CLR-Fc binding assay

NUNC maxisorb plates (Roskilde, Denmark) were coated with FhTE (125 μg/ml) overnight at 4 °C. Plates were blocked with 1% BSA in TSM (20 mM Tris, pH 7.4, 150 mM NaCl, 1 mM CaCl_2_ and 2 mM MgCl_2_), and 1 μg/ml of different hCLR-Fc in TSM were added for 2 h at room temperature. Specific binding was blocked through the pre-incubation of hCLR-Fc with the Ca^2+^-chelator EGTA (10 mM). For hMGL-Fc, the specific binding was blocked with free GalNAc (100 mM; Sigma-Aldrich) or blocking anti-hMGL antibody (1G6.6, 10 μg/ml), by pre-incubation for 30 min at 37 °C. Binding was detected using a peroxidase-labeled, anti-human IgG-Fc antibody (Jackson ImmunoResearch Laboratories, PA). Binding was visualized with 3,3′,5,5′-tetramethylbenzidine (TMB) as a substrate (Sigma Aldrich) and optical density was measured by spectrophotometry at 450 nm. When indicated, FhTE was pretreated with the following exoglycosidases: α-N-Acetylgalactosaminidase from chicken liver, α(1-2,3,4,6)-Fucosidase from bovine kidney or α(1-2,3,6)-Manosidase from jack bean (all Prozyme, CA), as indicated in manufacturer’s instructions. When the treatment was done with both Fucosidase and Mannosidase at the same time, a buffer sodium acetate 50 mM pH 5 was used.

### Internalization Assay

The internalization and binding of FhTE to mo-DCs was analyzed by flow cytometry. Briefly, mo-DCs (1 × 10^5^/well) were incubated with Alexa 647-labeled FhTE for 1 h at 37 °C in complete medium (to assess uptake), or at 4 °C in complete medium (to assess binding). Cells were then washed twice and the binding or internalization by CD11c^+^ cells was analyzed by flow cytometry.

### Imaging FACS

Mo-DCs were incubated with Alexa647-labeled FhTE for up to 1 h at 37 °C in complete medium, fixed and permeabilized. Then, they were stained with a DC-SIGN- (clone DCN46), LAMP-1- (clone H4A3) and EEA1- (clone 14/EEA1)-specific antibodies (from BD Biosciences) and analyzed by imaging FACS. Mo-DCs were acquired on the ImageStreamX (Amnis Corp.) as previously described. A minimum of 15,000 cells was acquired per sample at a flow rate ranging between 50 and 100 cells/s.

### Identification of N-Glycans

The identification of N-glycans present in FhTE was performed as previously described^36^,^37^. Briefly, 0,5 mg of FhTE was freeze dried, dissolved in 100 μL of buffer 0.25 M sodium phosphate buffer pH 8.5, containing 7 M urea, 2 M tiourea, 2% SDS, 1 M μ-mercaptoetanol and sonicated during one hour. Then 100 μL of buffer 0.25 M sodium phosphate buffer pH 8.5 containing 15% of Igepal CA-630 was added to neutralize the inhibitory effect of SDS on PNGase-F activity and was sonicated for another 15 min. Finally, 800 μL of 0.25 M sodium phosphate buffer pH 8.5 and 10 U of PNGase F were added and the mixture was incubated for at least 48 h at 37 °C. The purification of glycan structures was performed using PGC SPE columns of porous activated charcoal and the remaining detergent was removed by incubation with detergent out beads. Purified N-glycans were derivatized with 7-amino-4-methylcoumarin (AMC) via reductive amination in the reducing end. Briefly the oligosaccharides were mixed with 2 mg of AMC and 2 mg of reducing agent picoline borane, after thoroughly vortexing the mixture was incubated at 65 °C for 2 hours. Separation, quantification and characterization of glycans were performed using a 2D-LC-MS system with an intercalated nano-Fluorescence detector. As first dimension a capillary WAX trap column is used to trap the charged oligosaccharides species. Unbound neutral oligosaccharides were trapped onto a C18 trap column (300 μM × 10 mm, Dionex The Netherlands) and analyzed on a long reverse phase nano-LC column (5 μ, 75 μM × 3000 mm, prepared in-house). Glycan species were quantified based on fluorescence and structures were identified base on the information obtained from the MS/MS fragmentation pattern. Subsequently WAX bound charged species were eluted with 500 mM ammonium formate and trapped onto the C18 trap column and analyzed as mentioned above.

### Statistical analysis

Results were analyzed using one-way ANOVA (non-parametric Friedman test and Dunn’s post-test) or two-way ANOVA followed by Bonferrroni Multiple Comparison test using GraphPad Prism software (GraphPad Software, San Diego, CA). Results were considered to be significantly different when p < 0.05.

## Additional Information

**How to cite this article:** Rodríguez, E. *et al. Fasciola hepatica* glycoconjugates immuneregulate dendritic cells through the Dendritic Cell-Specific Intercellular adhesion molecule-3-Grabbing Non-integrin inducing T cell anergy. *Sci. Rep.* **7**, 46748; doi: 10.1038/srep46748 (2017).

**Publisher's note:** Springer Nature remains neutral with regard to jurisdictional claims in published maps and institutional affiliations.

## Supplementary Material

Supplementary Figure 1
