# Supplementary material for: Fasciola hepatica glycoconjugates immuneregulate dendritic cells through the Dendritic Cell-Specific Intercellular adhesion molecule-3-Grabbing Non-integrin inducing T cell anergy
Source: Sci Rep. 2017 Apr 24;7:46748. doi: 10.1038/srep46748 (PMC5402274; doi:10.1038/srep46748)
Supplement: Supplementary Figure 1 [file srep46748-s1.pdf]

Supplementary Information

Fasciola hepatica glycoconjugates immuneregulate dendritic cells through the Dendritic Cell-Specific Intercellular adhesion molecule-3-Grabbing Non-integrin inducing T cell anergy

Ernesto Rodríguez[a,#], Hakan Kalay[b], Verónica Noya[a], Natalie Brossard[a], Cecilia Giacomini[c], Yvette van Kooyk[b], Juan J. García-Vallejo[b] and Teresa Freire[a]

Laboratorio de Inmunomodulación y Desarrollo de Vacunas, Departamento de Inmunobiología, Facultad de Medicina, Universidad de La República, Montevideo, Uruguay[a]; Department of Molecular Cell Biology and Immunology, VU University Medical Center, Amsterdam, the Netherlands[b]; Laboratorio de Bioquímica, Departamento de Biociencias, Facultad de Química, UdelaR, Montevideo, Uruguay[c]

Supplementary Fig. 1

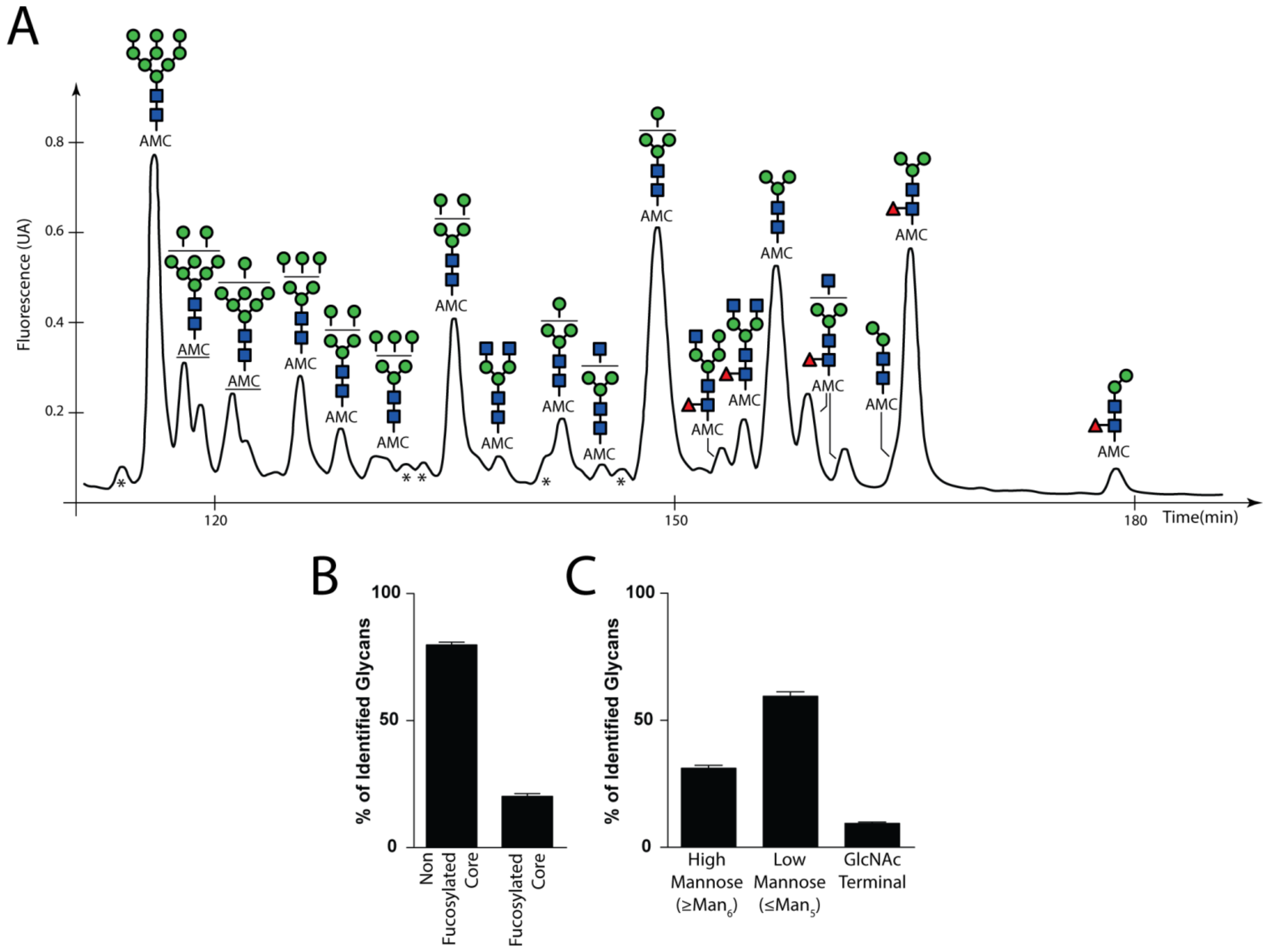

Supplementary Fig. 1. Identification of mannose and fucose-containing glycans by mass spectrometry. A) The N-glycans from 0,5 mg of FhTE were released, purified, AMC-labeled, and resolved by glycan nanoprofiling. The chromatography profile corresponding to the HPLC separation is shown with the corresponding structures identified by mass spectrometry. **B)** Quantification of fucosylated and non-fucosylated cores. **C)** Quantification of high-mannose, oligomannose and GlcNAc-terminal oligomannose N-glycans present in FhTE.
